# Development of a novel tool to assess therapists’ alliance through medical clinical reports in public mental health settings

**DOI:** 10.1192/j.eurpsy.2023.802

**Published:** 2023-07-19

**Authors:** A. Lewin, D. Tzur Bitan

**Affiliations:** Psychology, Ariel University, Ariel, Israel

## Abstract

**Introduction:**

Many studies demonstrate that monitoring of the therapeutic alliance can assist therapists to identify changes in patient-therapist relations and accommodate their interventions to prevent early termination. Nonetheless, therapists in public mental health institutes are only required to provide a textual description of patient’s visit, and are usually overloaded to complete empirical measures after each session.

**Objectives:**

The aim of this study is to develop a novel empirical tool to assess therapists’ perception of the therapeutic alliance through therapists’ clinical reports completed in public mental health.

**Methods:**

Patients (N=56) and their therapists (N=32) recruited to participate in a randomized controlled trial completed the Session Alliance Inventory- 6 items (SAI-6) after each session. In this measure, the working alliance included three components: an emotional bond, the agreement on goals, and the agreement on tasks. Afterwards, medical records were extracted and ranked using the SAI-6 by two independent researchers. Inter-rater reliability was .94, indicative of excellent reliability.

**Results:**

Overall, 163 sessions were extracted and evaluated, and were compared with 32 therapist evaluations and 56 patient evaluations. The factor structure of both coders demonstrated a two-factor solution explaining 89.38% of the variance for coder 1, and 71.50% of the variance for coder 2. For patients and therapists, a one-factor solution emerged, explaining 73.00% of the variance for patients, and 62.29% of the variance of the therapists. Both coders demonstrated higher factor loadings of the goals and tasks (0.75-0.81 for coder 1, 0.75-0.78 for coder 2) compared with the bond index (0.57-0.62 for coder 1, 0.52-0.56 for coder 2), indicating higher consistency across these subscales. Internal consistency was alpha Cronbach .87 for coder 1, 0.77 for coder 2, 0.92 for patients and 0.87 for therapists. The scale was partially associated with the therapists’ reports, with coder 2 having a stronger association with therapists ratings in all indexes (r= 0.04 -0.25) than coder 1 (r= 0.03- 0.15). Both coders did not correlate with therapists’ ratings on the bond component. As known in the scientific literature, patients rated the alliance higher than therapists (M=5.74, SD=1.36 for patients, and M=4.67, SD=0.84 for therapists, SE=0.10, p<0.001).

**Conclusions:**

The results of the current study demonstrate the feasibility of assessing therapists’ perceptions of the working alliance via therapists’ routine reports. The differences emerging in the factor structure suggests that coding the clinical reports in primarily beneficial for the evaluation of the agreement on the treatment goals and tasks, and less for the evaluation of patient-therapist emotional bond.

**Disclosure of Interest:**

None Declared

